# Phytobiotic-Prebiotic Feed Additive Containing a Combination of Carob Pulp, Chicory, and Fenugreek Improve Growth Performance, Carcass Traits, and Fecal Microbiota of Fattening Pigs

**DOI:** 10.3390/ani13233621

**Published:** 2023-11-23

**Authors:** Ákos Juhász, Viviána Molnár-Nagy, Zsófia Bata, Ko-Hua Tso, Katalin Posta

**Affiliations:** 1 Department of Microbiology and Applied Biotechnology, Institute of Genetics and Biotechnology, Hungarian University of Agriculture and Life Sciences, H-2100 Gödöllö, Hungary; posta.katalin@uni-mate.hu; 2 Dr. Bata Ltd., Bajcsy-Zs. u. 139, H-2364 Ócsa, Hungary; research@drbata.com (V.M.-N.); racheltso0331@gmail.com (K.-H.T.)

**Keywords:** carob pulp, chicory, fattening pigs, fecal microbiota, fenugreek, phytobiotic, prebiotic

## Abstract

**Simple Summary:**

The objective of this study is to examine the effects of a phytobiotic-prebiotic feed additive (PPFA) containing a combination of carob pulp, chicory, and fenugreek on growth performance, carcass traits, and fecal microbiota in fattening pigs. One kg/T of the PPFA shows a significant effect on growth promotion and a positive effect on lean meat production and carcass quality of fattening pigs. Moreover, the PPFA has the ability to regulate intestinal microbiota by improving beneficial bacteria while inhibiting some pathogen bacteria. This PPFA could be an effective antibiotic-free feed additive to enhance growth performances, improve carcass grade, and promote healthy gut development in fattening pigs.

**Abstract:**

The purpose of this study was to determine the effectiveness of a phytobiotic-prebiotic feed additive (PPFA, which contains a combination of chicory and extracts of carob pulp and fenugreek) in the diets of fattening pigs on growth indicators, carcass characteristics, and fecal microbiota. A total of 329 crossbred pigs were randomly divided into two dietary treatments, including a basal diet without additives as the control group and a basal diet supplemented with 1 kg/T PPFA as the trial group. The PPFA supplementation led to a significant increase in the body weight gain and average daily gain of the trial group compared to those of the control group after 70 days of feeding. Through the S-EUROP evaluation system, we also found that the fattening pigs fed PPFA significantly improved their carcass indicators. Furthermore, it was shown that PPFA regulated porcine intestinal microbiota, including promoting the growth of the beneficial commensal bacteria (i.e., *Bifidobacterium* and *Lactobacillus*) while inhibiting some potential pathogen bacteria (i.e., *Bacteroidaceae* and *Campylobacteraceae*). Our work revealed that the phytobiotic-prebiotic feed additive containing carob pulp, chicory, and fenugreek positively influences the intestinal microbiota, growth performance, and carcass traits in fattening swine.

## 1. Introduction

The fattening phase can directly affect lean meat deposition and the uniformity of carcasses at production [1]; therefore, it is an essential phase in the pig growth stage. During the fattening phase, swine dysentery is another problem [2]; thus, antibiotic growth promoters (AGPs) are commonly used as feed additives to avoid this issue and to increase animal performance and infection resistance [3]. However, AGPs induce the development and increase of antibiotic-resistant bacteria [4]. Antibiotic growth promoters create severe threats to the health of humans and animals. Due to the above reasons, many countries have banned AGPs for environmental and public health concerns [5]. Thus, it is necessary and urgent to find effective alternatives for AGPs. Among the commercially existing alternatives, prebiotics and phytobiotics have been reported to enhance the performance and health of pigs [6,7].

Prebiotics, such as carob pulp and chicory, are mainly complex carbohydrates that serve as substrates to the gut microbiome, thereby selectively stimulating the growth and activity of specific core or more-dominant gut bacteria [8,9]. Carob pulp is a mixture of macro- and micronutrients, such as carbohydrates, vitamins, and minerals, and secondary metabolites (such as tannins) with functional properties [10]. Highly polymerized condensed tannins of carob pulp are a heterogeneous group of phenolic compounds which have been shown to exert positive effects on the functionality of the gastrointestinal tract, growth performance, and meat quality in swine [11,12,13]. Carob pulp has been suggested to prevent porcine diarrhea [12]. On the other hand, chicory contains a high content of inulin and fructooligosaccharides, creating its potential prebiotic capacity [14]. Previous research indicated that chicory influenced the intestinal microenvironment of pigs; for instance, it increased the number of lactic acid bacteria in the distal ileum [14,15]. Furthermore, inulin not only improved body weight gain but also significantly increased the dressing percentage and tended to increase the loin-eye area, showing the beneficial effect of inulin supplementation on the growth performance and carcass traits in growing-finishing pigs [16].

Phytobiotics, including plant extracts and compounds, have also been studied as an alternative to AGPs due to their antimicrobial, antioxidant, and anti-inflammatory activities and their beneficial influence on animal gut function and performance in swine [17,18]. Phytobiotics have the potential to inhibit pathogenic bacteria and show a range of host-related responses, such as improvement in beneficial intestinal microbiota and the digestive or immune function of the host [19,20,21]. The carbohydrate fraction and the polyphenol of fenugreek seed are the essential parts that can modulate changes in the intestinal ecology, making a higher differentiation of the microbiota in the porcine caecum [22,23]. Previous research indicated that the piglets fed on a fenugreek diet had higher *Lactobacillus* and lower *Escherichia*, *Hafnia*, and *Shigella* abundance in the small intestine [22]. In addition, the dietary supplementation of fenugreek seed improves growth performance, digestibility, and immunity in growing pigs [24].

Our previous study demonstrated that a phytobiotic-prebiotic feed additive (PPFA) might be an effective AGP alternative for preventing post-weaning diarrhea and promoting the development of a balanced gut system [25]. However, fattening pigs showed higher microbial richness than weaning and growing pigs, which is attributable to their microbial community’s higher maturity and stability [26]. For this reason, growth performance and intestinal changes may differ between piglets and finishing pigs. Thus, this study aimed to investigate whether combinations of carob pulp, chicory, and fenugreek seed produce a possible synergy effect on the growth performance, carcass traits, and fecal microbiome in fattening pigs.

## 2. Materials and Methods

### 2.1. Animal Management and Experimental Design

This large-scale field experiment was performed at the commercial farm of Dunahyb Kft. (Fadd, Hungary). A total of 329 crossbred (Topigs Norsvin TN70 × DanBred) pigs with mixed gender were involved in the treatment, with a starting average body weight of 32.7 ± 2.1 kg (Table S1). Pigs were ear-tagged individually using a plastic tag and were randomly allocated into two trial groups: control group (C) only containing basal diets and trial group (T) containing basal diets supplemented with 1 kg/T of PPFA. The PPFA was provided as a commercial feed additive by Dr. Bata Ltd. (Budapest, Hungary) and it contained a combination of chicory and extracts of carob pulp and fenugreek (Table 1). All pigs received a two-phased diet: Grower I from day 0 to day 42, and Grower II from day 42 to the end of the trial (day 70). All diets were calculated to be nutritionally equivalent and to meet the nutrient requirements recommended for the crossbred species. The compositions of the basal diets for the two phases are presented in Table 2.

The mashed pig feed was provided ad libitum throughout the trial from hoppers and water from nipple drinkers per pen. The feed intake of each group for both Grower I and Grower II was recorded daily, and the total feed consumption for the whole experiment was calculated. All fattening pigs were measured individually and manually at the beginning (day 0) and the end of the trial (day 70). Body weight gains were calculated for the 70-day trial period based on the measured finishing and starting body weights. The feed conversion ratio (FCR) was calculated based on the total consumed feed per pen and the average body weight gain of the pen.

All pigs were placed in same-sized pens. Stocking densities complied with the European Union (EU) Council Directive [28], which set down minimum standards for protecting pigs. Both treatment groups involved 10 pens, each containing 15–18 pigs. The pigs were housed in a barn lit only by daylight. The daily light was maintained for 15–16 h during the trial period. The caretakers monitored health status daily, and no illness was observed.

### 2.2. Meat Sampling and Carcass Classification

At the end of the trial period, all pigs were conventionally slaughtered by a local slaughterhouse and chilled overnight. At a 24 h postmortem, the longissimus muscle from the left side between the 5th and 13th rib was removed. Then, carcass qualities were evaluated using the S-EUROP system. The S-EUROP system is used to classify carcasses according to their shape and fat level on an alphanumeric (S-P) scale for confirmation class and a numerical (1–5) scale for fat class [29]. This system evaluates six main criteria: visual fat appearance; tissue quality; muscularity; conformation; carcass length; and overall carcass area. These criteria are scored on a scale of 1 to 5. Scores are given for each criterion, factored in, and then averaged for the total score of the carcass [30]. The visibility of fat is determined by examining the coverage and distribution of fat on either side of the midline. Tissue quality is evaluated by looking at intramuscular fat content and color. Muscularity is assessed through observation of carcass shape and contour. Carcass length and width are measured from neck to tail and from shoulder to pelvic inlet, respectively. Conformation is evaluated based on how close the measured width and length are to the ideal ratio of width/length. Finally, the overall carcass area is noted and factored into the final score.

### 2.3. Fecal Sample Analysis: Enumeration, DNA Extraction and Sequencing

One fecal sample per pen was collected before the start of the experiment (S: start samples) and 70 days later at the end of the feeding trial (T: trial and C: control samples). The samples were collected during the peak hours of defecation in sterile fecal containers (Biolab Inc., Budapest, Hungary) from the pen floor, and the floor was cleaned before and after sampling to avoid contamination.

The detailed protocols of sample handling, colony forming unit (CFU) determination, DNA extraction, and sequencing were published earlier [31]. Briefly, the fecal samples were homogenized, diluted, and plated in duplicate on different culture media: De Man, Rogosa and Sharpe Agar, Tryptose Sulfite Cycloserine Agar, Eosin Methylene Blue Agar, Nutrient Agar, and Columbia Blood Agar were used in case of lactic acid bacteria (LAB), *Clostridium perfringens*, coliform-, total aerobic, and anaerobic bacteria, respectively. All media were purchased from Biolab Inc. (Budapest, Hungary). The inoculated agar plates were incubated under conditions suitable for each microbial group. The number of viable bacteria were calculated per gram of feces (CFU/g feces, wet weight). The results are presented as log_10_-transformed data.

The DNA was extracted from the fecal samples (S, C, and T groups) using Quick-DNA Fecal/Soil Microbe Microprep Kit (ZYMO Research, Irvine, CA, USA). The abundance of the bacterial communities of the fecal samples were estimated using high-throughput sequencing of the V3–V4 region of the 16S rRNA gene on the Illumina MiSeq platform at UD-GenoMed Ltd. (Debrecen, Hungary).

### 2.4. Bioinformatics

The 16S rRNA gene paired-end amplicon reads were processed using the Frogs pipeline [31]. Briefly, forward and reverse reads were filtered and merged using VSEARCH [32] with the following parameters: min amplicon size: 44; max amplicon size: 550; and mismatch rate: 0.1. Merged sequences were clustered using Swarm [33]. Chimeric sequences were removed using *remove_chimera.py* of the Frogs pipeline, then the sequences were grouped into clusters and filtered: all clusters containing fewer sequences than 0.005% of all sequences were removed from further analysis. Taxonomic assignment was performed using BLAST [34] against Silva Database 138.1 [35] for ribosomal small-subunit RNA. Finally, the information pertaining to Amplicon Sequence Variant (ASV) abundance was normalized using a standard sequence number corresponding to the sample with the least number of sequences. Subsequent analyses were performed based on the normalized data. The alpha diversity indices (and all other diversity analyses) were calculated using the FROGS Phyloseq v1.38.0 tool [36]. Microbial diversity was visualized using multidimensional scaling (MDS). Permutational multivariate ANOVA (PERMANOVA) was performed to estimate the amount of variation explained by treatments and sampling times with 9999 permutations. The analysis of differential abundance of C and T groups was performed using the DESeq2 package [37]. Linear discriminant analysis (LDA) coupled with the linear discriminant analysis effect size (LEfSe) algorithm was also used to identify biomarkers as a characteristic of each group based on the abundance values [38].

### 2.5. Statistical Analysis

For the statistical evaluation of the efficacy of the additive, generalized linear mixed model (GLMM) analysis of variance (ANOVA or Welch ANOVA) design was applied. In the statistical analysis the dependent variable (response variable) was the measured end parameter, and the categorical factors were the treatments (control and trial). For evaluating all production end-parameters, the experimental unit was taken to be the pen, as the smallest entity to which the treatments were applied [39].

Statistical analysis of CFU values and alpha diversity indices were performed with R Statistical Software 4.0.4 [40]. Values were expressed as means ± SD. Differences between treatments were determined by ANOVA, followed by Tukey’s post-hoc test. In all tests, a *p* < 0.05 was considered to indicate statistical significance.

## 3. Results

### 3.1. Growth Performance

First, the effect of feeding the PPFA (1 kg/T) on the growth parameters (Table 3) of pigs was evaluated and compared to the control group. There were no statistically significant differences in the body weight at 0-day between C and T groups. The body weight and average daily gain (ADG) of the pigs in the trial group were higher than the body weight and ADG of the control pigs at 70 days; however, there were no significant differences in the FCR.

### 3.2. Carcass Quality

Our results (Table 4) reported that the average lean meat percentage in PPFA-treated fattening pigs was significantly (*p* < 0.0001) higher than that of non-treated fattening pigs. According to S-EUROP results, the PPFA-treated group had more high-quality S-grade carcasses than the control group. In contrast to the control group, the PPFA-treated group did not have lower-quality R- and O-grade carcasses.

### 3.3. Fecal Microbiota Composition

#### 3.3.1. Enumeration

The effect of different treatments on the intestinal microbiota composition of the fattening pigs was characterized by cultivation-dependent and independent methods from excreted faces. First the amounts of LAB, *C. perfringens*, coliform-, aerobic- and anaerobic bacteria were determined using different selective media (Table 5). The S samples (both groups) were collected before the treatments were applied and the C and T samples (C and T groups) were collected at the end of the experiment. All pigs included in the experiment were healthy, which is supported by the low colony count of coliform bacteria (less than 10^6^ CFU/g) and *C. perfringens* (around 10^4^ CFU/g).

There was no significant difference between the feces of S, C, and T groups in the amount of coliform and anaerobic bacteria and *C. perfringens*. The number of aerobic bacteria was significantly lower (*p* < 0.001) in the case of S than C or T, but there were no significant differences between C and T. Among the five microbial groups examined, only LAB showed a significant difference between groups T and C: the amount of LAB was significantly (*p* < 0.05) higher in T than C. The mean values of LAB in the feces of C and S groups were similar.

#### 3.3.2. Sequencing

In order to investigate the effect of PPFA on the fecal bacterial composition in fattening pigs, 16S rDNA sequencing was conducted on all samples. Illumina MiSeq sequencing generated 3,139,800 sequences from the fecal samples. The sequences were filtered, grouped into clusters, and identified based on 97% species similarity. Finally, 1,730,925 sequences grouped into 753 ASVs were kept and analyzed. The number of ASVs per sample ranged from 653 to 715. The average ASV number of S samples (665.0 ± 7.17) were significantly (*p* < 0.001) lower compared to those of C (706.0 ± 2.55) and T (705.6 ± 6.07) samples. The numbers of identified species (*p* < 0.001), genera (*p* < 0.001), and families (*p* < 0.05) were also significantly less in S than in C or T samples. However, C and T samples had no significant difference (Table S2). All individual samples shared 501 ASVs, and all ASVs were present in at least three different samples. Although no sample-specific ASVs existed, eight unique ASVs were identified in S samples when comparing the in silico merged data of S, C, and T samples (Figure S1).

#### 3.3.3. Taxonomic Profiles and Bacterial Diversity

**Figure 1:**
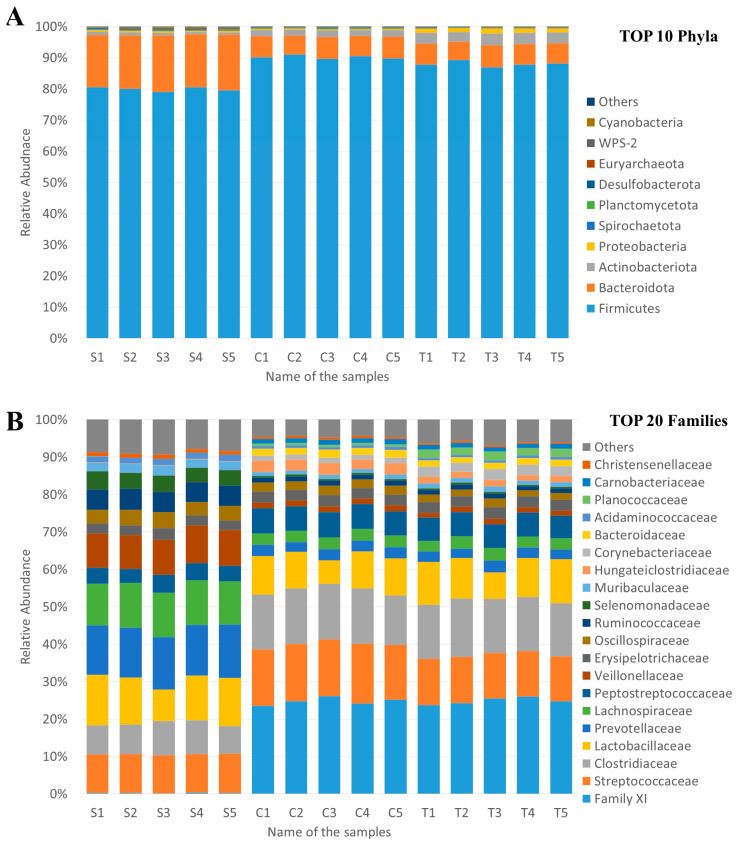
Relative abundance of bacterial community of the fecal samples. (**A**) Top 10 phyla. (**B**) Top 20 families.

A total of 14 phyla, 19 classes, 43 orders, 73 families, 199 genera, and 276 species were detected by sequencing. The three most abundant phyla in all samples were Firmicutes, Bacteroidota and Actinobacteriota with an average abundance of 86.07%, 10.19%, and 2.13%, respectively (Figure 1). The average abundance of all other identified phyla was less than 1%, but even at the phylum level there were significant differences between S, C, and T samples. The average abundance of Firmicutes was 79.98% in the case of S samples, but it was significantly (*p* < 0.001) higher in C (90.24%) and T (87.99%) samples. The average relative abundance of Bacteroidota showed the opposite trend: it was 17.34% in the case of S samples, but only 6.66% in C and 6.56% in T samples. The average relative abundance of Actinobacteriota was 0.89% (S), 2.01% (C), and 3.50% (T). The fourth most abundant phyla in all samples were Proteobacteria (0.75%), but the relative abundance of this phylum was 1.38% in T samples and less than 0.5% in all other samples.

The phylogenetic classification of samples showed greater (mainly sampling-time-based) differences at lower taxonomy ranks. For example, at the family level, the most abundant family and its average abundance was *Family XI* (16.62%), but it was only 0.25% in S samples and 24.74% and 24.87% in C and T samples, respectively.

**Figure 2:**
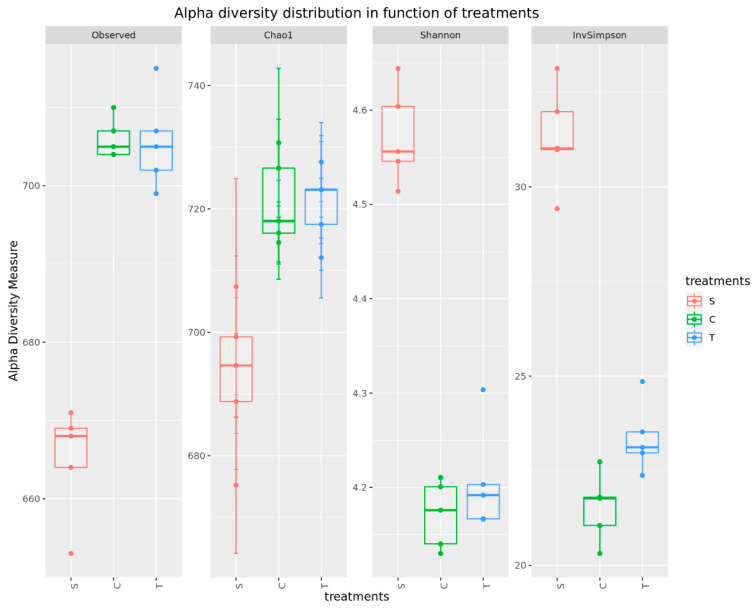
Alpha diversity indices of fecal samples grouped by treatments. All alpha diversity indices of S samples were significantly (*p* < 0.001) different from the values of C and T samples. There was no significant difference between the C and T samples.

The analysis of bacterial diversity also confirmed the significant difference of the S samples from the C and T samples. Alpha diversity indices, shown in Figure 2 and Table S3, showed main differences by sampling time. The observed species (*p* < 0.001) and Chao1 (*p* < 0.001) were significantly lower, but Shannon (*p* < 0.001) and invSimpson (*p* < 0.001) were significantly higher in S samples than C and T samples, which suggests that the diversity of samples was higher at the beginning of the experiment (S) than at the end (C and T), despite fewer identified species. The invSimpson index was significantly lower in C than T samples (*p* < 0.05), and the other examined alpha diversity indices were close to the same.

**Figure 3:**
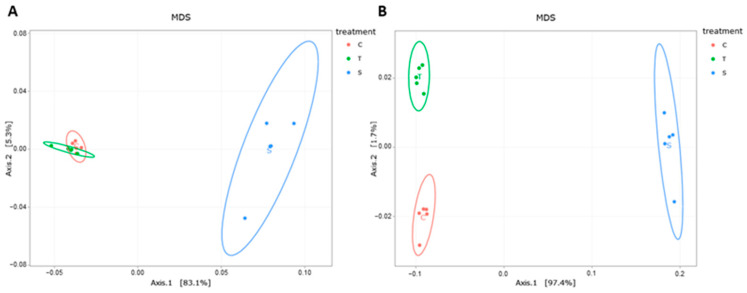
Beta-diversity of the fecal samples. Multiple dimension scale (MDS) plots of unweighted (**A**) and weighted (**B**) UniFrac distances of the microbial communities of the fecal samples.

The beta-diversity analysis confirmed the greater distance between S and C or T samples, and also confirmed that the ASV content of C and T samples were very similar, but the ASV abundance was slightly different (Figure 3). PERMANOVA indicated that the sampling time (S sample for 0 day as well as C and T samples for 70 day) was a stronger driver of the community structure, explaining 97.31% of the variation (*p* < 0.001) than treatments (C vs. T, 70.91%, *p* < 0.001) based on weighted UniFrac distances.

#### 3.3.4. Treatment-Related Differences

**Figure 4:**
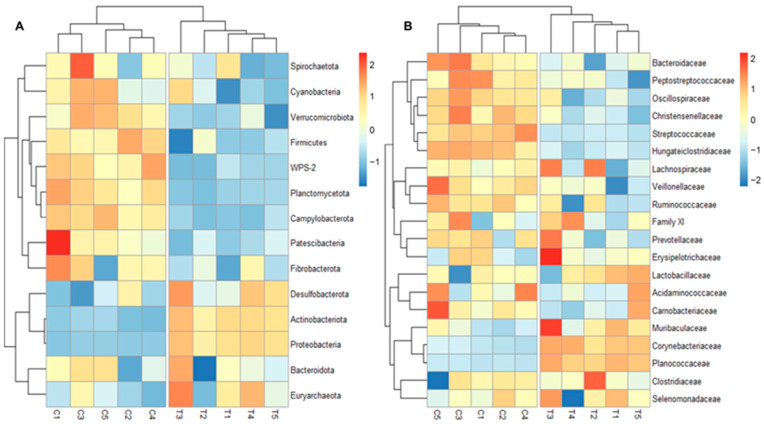
Heatmap of all phyla (**A**) and the 20 most abundant families (**B**) of bacteria of control and trial fecal samples. The color scale shows the Z-score of abundance of families within each group.

The taxonomic composition of the microbiota of the fecal samples and the results of the bacterial diversity analyses primarily indicate that the most significant changes can be linked to the sampling times (to the age of the pigs). Age-related shift of the intestinal microbiota is well known and has been thoroughly studied. Therefore, we chose to focus on analyzing changes of microbiota caused by PPFA compared to the controls (Figure 4).

The most abundant phyla (Firmicutes) showed small but significant differences at the phylum level. It was significantly (*p* < 0.001) higher in C (90.24%) than in T (87.99%) samples. Two of the three most significantly differed phyla were more abundant in C than T (Campylobacterota 8.6-fold and WPS-2 3.2-fold), while Proteobacteria was more abundant in T (1.38%) than C (0.36%).

Among the top 20 most abundant families, *Planococcaceae* showed the biggest difference, with an average abundance of 0.59% in C and 2.14% in T samples. *Lactobacillaceae* (C: 9.20%, T: 10.31%), *Corynebacteriaceae* (C: 1.34%, T: 2.68%), and *Muribaculaceae* (C: 1.12%, T: 1.28%) were also more abundant in trial samples, while the average abundances were higher in control samples in the case of *Streptococcaceae* (C: 15.27%, T: 12.20%), *Veillonellaceae* (C: 1.54%, T:1.39%), *Oscillospiraceae* (C: 2.38%, T:1.86%), *Hungateiclostridiaceae* (C: 2.92%, T: 1.68%), *Bacteroidaceae* (C: 1.95%, T: 1.62%), and *Christensenellaceae* (0.57%, T: 0.44%), but most of these changes were less than two-fold. Greater differences were observed in the case of less abundant families, for example *Campylobacteraceae* was 8.6-fold more abundant in C than T, but the average abundance was only 0.022% and 0.003%, respectively. More-detailed differential abundance analysis of ASVs was performed with DESeq2, and it revealed that the abundance of 377 of 753 ASVs differed significantly (*p* < 0.05): 191 ASVs were more abundant in C, and 186 were more abundant in T samples.

For those ASVs that were significantly more abundant in C than T samples, the greatest difference (log_2_-fold change) was observed in the case of *Hungateiclostridiaceae*, *Family XI* and *Oscillospiraceae* (Figure S2). Among the clusters occurring in significantly higher abundances in T samples, the biggest log_2_-fold change was in the case of *Moraxellaceae*, *Planococcaceae* (*Savagea* and *Lysinibacillus*), and *Lachnospiraceae*. This result was confirmed with LEfSe: the C related features were *Hungateiclostridiaceae* and *Streptococcaceae*, while T related features were *Moraxellaceae*, *Planococcaceae*, and *Corynebacteriaceae*.

We also observed changes that were less significant or indicated microbes whose mean abundance was smaller compared to those previously mentioned, but which may be more important in terms of understanding the mode of action of the feed supplement. Some pathogen microbes were more abundant in feces of C than T groups: *Campylobacter* (C: 0.022%, T: 0.003%, *p* < 0.001) and *Chlamydia* (C: 0.002%, T: 0.0015%, *p* < 0.05), while some potentially probiotic microbes were more abundant in T than C groups: *Lactobacillus* (C: 5.22%, T: 6.43, *p* < 0.05) and *Bifidobacterium* (C: 0.13%, T: 0.16%, *p* < 0.05).

## 4. Discussion

The investigation of gut microbial composition and variability is significant for animal health, growth performance, and carcass quality, given the importance of gut microbiota [41]. Long-term misuse and abuse of antibiotics have led to gut dysbiosis, induction of antibiotic resistance and antibiotic resistance genes in pathogenic bacteria, and antibiotic residue in porcine edible tissues, raising public concerns over food safety, antibiotic ineffectiveness, and the spreading of zoonotic bacteria [42,43,44]. Therefore, many natural sources of feed additives in livestock husbandry are emerging as attractive alternatives to antibiotics, such as phytogenics, probiotics, and prebiotics [45,46,47,48]. In the present study, we investigated the effects of a PPFA on the growth performance and carcass traits of finishing pigs and further used the results of the traditional culture-based microbiological and modern metagenomic analysis to verify its correlation.

In growth performance, our results showed that the PPFA supplementation significantly (*p* ≤ 0.001) increased ADG and final body weight (+1.72% or +1.8 kg per pig on average) compared to the control group. There were no significant differences in ADFI and FCR was slightly, but not significantly, lower for T than C groups. In conclusion, the T group receiving the feed supplement performed better than the C group regarding FCR. In carcass quality, we found that PPFA-treated pigs (59.5%) had higher lean production than the control group (57.2%). In addition, the T group (50.9%) had more high-grade (S grade) carcasses than the C group (20.5%). These results agree with previous literature for the individual ingredients, chicory, fenugreek, and carob pulp. As a high-efficiency prebiotic, inulin (the main active agent of chicory) improving growth performance, carcass traits, and the meat quality of pigs has been reported in numerous studies [16,49]. For instance, the ADFI and ADG of weaned piglets fed with 2.5 kg/T inulin increased by 12.2 and 20.1%, respectively [50]. Further, 0.5% inulin increased ADG by 9% and 6% for growing (32–70 day) and finishing (70–90 day) pigs, respectively. For the carcass traits, compared to the control group, 5% inulin-treated pigs improved carcass weight by 7.98% and dressing percentage by 3.85% [16]. Many health-promoting properties of inulin interventions have been attributed to enhancing saccharolytic fermentation and resulting in increased production of short-chain fatty acids (SCFAs) by the colon microbiota, thereby inhibiting pathogenic microbes and improving the homeostasis of gastrointestinal tracts [14,51,52]. Various studies have shown that dietary supplementation with fenugreek seed extract improves growth performance, nutrient digestibility, and immune status in sows and weaning pigs [22,24,53,54]. For instance, a diet containing 0.2% fenugreek seed improved ADG by 4.2% in growing pigs [24]. In addition, the administration of 0.2% fenugreek seed extract has been reported to reduce fecal pathogen bacterial (*E. coli)* counts in sows by 9% [55]. A previous report showed that the dietary addition of 100 g/kg carob pods increased final body weight by 3.5% and carcass weight by 3.6% in fattening pigs and also improved the pork’s fatty acid content [13].

Fecal microbiota influences intestinal nutrient utilization, which affects growth performance and carcass traits in pigs, which have been investigated in many studies [56,57]. To better understand the effect of the PPFA on the gut microbial community and how the gut microbiota further influence the growth performance and carcass traits in fattening pigs, the culture-based broad microbial counts were complemented with a highly detailed metagenomic analysis of the fecal samples [25]. In agreement with our results, many studies [58,59] have reported that Firmicutes and Bacteroidetes were the two most abundant families in all porcine fecal samples. The average abundance of Firmicutes in the T and C group samples (the samples were collected at the end of the trial) was significantly higher than that in the S group samples (the samples were collected at the beginning of the trial from both groups), but Bacteroidetes presented the opposite trend. In addition, similar to the results of Oh et al. [58], the abundances of two major phyla, Firmicutes and Bacteroidetes, did not significantly differ between the high body weight (as T group in our research) and low body weight (as C group in our research) groups, but some genera showed significant differences. Previous research reveals that some “core” bacterial genera, including *Bacteroides*, *Prevotella*, and *Lactobacillus*, can be found in more than 90% of healthy pigs at different ages [60]. Adding substrates preferred by these target microbes will help to increase the abundance of specific symbiotic species and benefit the gut health of pigs.

We demonstrated that the abundance of several taxa (*Bifidobacterium*, *Lactobacillus*, *Lachnospiraceae*, and *Muribaculaceae*) was increased in the PPFA-treated pigs. Several species within some of these genera (e.g., *Bifidobacterium* and *Lactobacillus*) are often employed as probiotics and in producing feed additives to prevent diarrhea or improve growth in pigs [61]. Therefore, a diet containing PPFA can potentially enhance the porcine fecal microbiota. Several studies have also found similar results, in that the abundance of *Bifidobacterium*, *Lactobacillus*, *Lachnospiraceae*, and *Muribaculaceae* increased, while those of *Campylobacter*, *Chlamydia*, and *Treponema* decreased, in chicory-treated pigs [62,63]. These studies support the idea that the inulin of chicory mitigated disorders in gut microbiota by enhancing saccharolytic fermentation, therefore promoting the growth of SCFAs (acetic, lactic, and propionic acids) and producing beneficial intestinal bacteria (such as *Bifidobacteria, Lactobacilli*, and *Muribaculaceae*) while inhibiting pathogen intestinal bacteria (e.g., *Bacteroidaceae* and *Campylobacteraceae*) [64,65,66]. These beneficial commensal intestinal bacteria (e.g., *Lactobacillaceae* and *Bifidobacterium*) improve the absorption of nutrients and produce a healthier intestinal system [67]. In addition, the abundance of intestinal microbiota limits pathogens. such as *Salmonella* infection, a mechanism referred to as colonization resistance [68].

In this research, we also found that the abundance (observed species and Chao1 index) of C group and T group samples significantly increased compared to that of S samples. This phenomenon means the abundance of intestinal microbiota becomes more complex with age in pigs [69]. Fattening pigs showed higher microbial richness than weaning and growing pigs, which is attributable to the higher maturity and stability of their microbial community [26]. However, the diversity (Shannon and invSimpson) of the S samples was higher than that of the C and T group samples.

The correlation between porcine gut microbiome and animal health during critical growth stages has been characterized in several studies [70,71]. Bacterial taxa such as *Christensenellaceae* and *Lactobacillus* have been positively related to body weight gain and feed efficiency, which is critical for the swine industry [55,57,72]. Hence, a healthy intestinal microbiota has an essential role in pig production. In our case, the CFU of LAB was significantly (*p* < 0.05) higher in the T than C samples. The metagenomic analysis also confirmed that the abundance of *Lactobacillus* (C: 5.22%, T: 6.43%) and *Lactobacillaceae* (C: 9.20%, T: 10.31%) in T samples was approximately 1% higher than in C samples. However, this difference proved to be insignificant. The abundance of *Christensenellaceae* and *Oscillibacter* did not differ significantly. On the contrary, porcine growth performance has also been confirmed to negatively correlate with some endogenous bacteria, such as *Bacteroidaceae*, *Campylobacteraceae*, *Corynebacterium*, and *Treponema* [57,73,74]. In our case, *Bacteroidaceae* (*p* < 0.01) and *Campylobacteraceae* (*p* < 0.001) were significantly decreased and *Treponema* was slightly lesser in T than C groups, although *Corynebacterium* was more abundant in T samples. It should be noted that each pig was healthy in both treatment groups. This is in agreement with the finding that potentially pathogenic bacteria abundance was low. The genus *Corynebacterium*, which currently has more than 110 validated species, is highly diversified [75]. Although some species belonging to the *Corynebacterium* genus are potential opportunistic pathogens [76], some species are non-pathogenic bacteria and are widely used in food and amino acid production, such as *C. callunae, C. casei*, and *C. crenatum* [75,77].

Intestine and fecal microbiota composition not only affects growth performance but also influences the carcass traits in pigs [73]. In particular, evidence is emerging for associating *Lactobacillus* in the gut or feces with a leaner phenotype and/or improved feed efficiency [57]. In agreement with previous research, our results indicated that PPFA-treated pigs had a higher abundance of *Lactobacillus* (C group: 9.20%; T group: 10.31%) and lean meat percentages (C group: 59.5%; T group: 57.2%) compared to the control group. For carcass quality, S-EUROP indices suggested that the PPFA-treated group performed better than the control group.

## 5. Conclusions

In the present study, we have demonstrated that the 1 kg/T of PPFA containing a combination of carob pulp, chicory, and fenugreek effectively improves growth performance, carcass traits, and fecal microbiota in fattening pigs. The PPFA was able to control the amount of potentially pathogenic bacteria (e.g., *Bacteroidaceae* and *Campylobacteraceae*) and increase the number of beneficial microbes (e.g., *Bifidobacterium* and *Lactobacillus*). The intestinal bacteria distribution became enriched in pigs treated with PPFA, which also improved body weight gain and increased the lean meat percentage and carcass grade. For practical feeding and management of fattening pigs, using this PPFA as their dietary supplement can be an optimal solution for both antibiotic-free intestinal regulation and a growth-promoting feed additive.

## Figures and Tables

**Table 1:** Chemical compositions of phytobiotic-prebiotic feed additive.

Ingredient ^1^	
Chicory (*Cichorium intybus* L.) roots ^1^, %	50.0
Carob (*Ceratonia siliqua*) seed, %	30.0
Fenugreek (*Trigonella foenum-graecum*) seed, %	20.0
Total, %	100.0
Calculated nutrient composition	
Dry matter, %	88.0
Crude protein, %	10.0
Crude fiber, %	20.0
Main active compound composition	
Inulin ^2^, %	2.5
Total polyphenolic compounds, GAE ^3^/g	40.0

^1^ European Union Register of Feed Materials number: chicory-4.4.1; Carob-3.2.7; fenugreek seed-3.5.1. ^2^ Inulin content analysis is according to AOAC 999.03. ^3^ GAE: gallic acid equivalent, the analysis method is according to ISO 14502-01:2005 [27].

**Table 2:** Ingredients and nutrient compositions of the basal diet (as fed basis).

Ingredient	%			
	Grower I (Day 0–42)		Grower II (Day 42–70)	
Barley	10.5		8.5	
Triticale	25.0		32.0	
Corn	36.5		35.0	
Malt germ, 26%	4.0		6.0	
Soybean meal, 46%	9.0		4.5	
Extracted rapeseed meal	0.0		5.0	
Pressed linseed	3.0		0.0	
Full fat soy	9.0		6.0	
^1^ Premix	3.0		3.0	
Total	100.0		100.0	
Calculated nutrient composition	^2^ C	^3^ T	C	T
Dry matter, %	89.9	89.4	89.2	89.6
Crude protein, %	15.0	15.1	14.2	14.4
Crude fiber, %	5.27	4.82	5.41	5.36
Crude fat, %	3.45	3.61	2.77	2.93

^1^ Supplied per kg of premix: vitamin A 150 IU; vitamin D_3_ 33.5 IU; vitamin E 2.5 mg; iron 5.201 mg; copper 450 mg; manganese 2.498 mg; zinc 1.50 mg; iodine 129 mg; selenium 14.04 mg. ^2^ C: control diet only contained basal diet. ^3^ T: trial diet contained basal diet supplemented with 1 kg/T of PPFA.

**Table 3:** Effects of PPFA on the growth performance of fattening pigs.

Item	C Group	T Group	*p* Value
Initial body weight, kg	32.8 ± 0.5	32.7 ± 0.5	0.6220
Final body weight, kg	104.9 ± 1.6 ^b^	106.7 ± 1.7 ^a^	0.0010
Body weight gain, kg	72.1 ± 1.5 ^b^	74.0 ± 1.7 ^a^	0.0001
ADFI, g/pig/day	2780.3 ± 90.9	2835.4 ± 90.3	0.1900
ADG, g/pig/day	1030.3 ± 16.0 ^b^	1057.8 ± 10.8 ^a^	0.0001
FCR	2.70 ± 0.07	2.68 ± 0.09	0.6350

^a,b^ Means ± SD with different superscripts in the same row differ significantly (*p* < 0.05). ADFI: average daily feed intake; ADG: average daily weight gain; FCR: feed conversion ratio.

**Table 4:** Effects of PPFA on lean meat (means ± SD) and S-EUROP system carcass classifications of fattening pigs.

Variable	C Group	T Group
Lean meat, %	57.2 ± 0.7 ^b^	59.5 ± 0.7 ^a^
% of cases in S class	20.5	50.9
% of cases in E class	58.4	44.0
% of cases in U class	17.5	5.0
% of cases in R class	2.4	0.0
% of cases in O class	1.2	0.0
% of cases in P class	0.0	0.0

^a,b^ Means with different superscripts in the same row differ significantly (*p* < 0.0001).

**Table 5:** Fecal microbiota composition of pigs with different diets.

Item, log_10_ CFU/g	S Group	C Group	T Group
Aerobic bacteria	6.72 ± 0.73 ^a^	8.06 ± 0.46 ^b^	7.96 ± 0.75 ^b^
Anaerobic bacteria	9.33 ± 0.44 ^a^	9.39 ± 0.26 ^a^	9.39 ± 0.29 ^a^
Lactic acid bacteria	8.97 ± 0.32 ^a^	8.92 ± 0.31 ^a^	9.27 ± 0.18 ^b^
Coliform bacteria	5.93 ± 0.75 ^a^	5.39 ± 0.49 ^a^	5.41 ± 0.54 ^a^
*Clostridium perfringens*	4.05 ± 0.61 ^a^	4.26 ± 0.48 ^a^	4.27 ± 0.51 ^a^

The number of samples was *n* = 10 (per treatment). Means with different superscripts in the same row differ significantly (*p* < 0.05). Significance between mean ± SD was determined separately for each microbial group. S group: the samples were collected at the start of the trial before applying any treatments. C groups: the samples were collected from the control group at the end of the trial; T groups: the samples were collected from the trial group at the end of the trial.

## Data Availability

Data is contained with the article or Supplementary Materials.

## Supplementary Materials

The following supporting information can be downloaded at: https://www.mdpi.com/article/10.3390/ani13233621/s1, Table S1. Arrangements for the experimental pigs. Table S2. Base sequence data information of all samples; Figure S1. Venn diagram showing the number of unique and shared ASVs of in silico merged data of treatments; Table S3. Alpha diversity indices of all samples and treatment merged groups. Figure S2. Significantly different abundant taxa between the control (C) and trial (T) groups.
