# Supplementary material for: Phytobiotic-Prebiotic Feed Additive Containing a Combination of Carob Pulp, Chicory, and Fenugreek Improve Growth Performance, Carcass Traits, and Fecal Microbiota of Fattening Pigs
Source: Animals (Basel). 2023 Nov 23;13(23):3621. doi: 10.3390/ani13233621 (PMC10705162; doi:10.3390/ani13233621)
Supplement: Supplementary file 1 [file animals-13-03621-s001.zip › animals-2701440-supplementary.pdf]

animals

MDPI

Table S1. Arrangements for the experimental pigs.

Item	C group	T group
Initial body weight (kg)/per pig	32.8 ± 0.4	32.7 ± 0.5
Trial period (day)	70	70
Replicates (pens) per group	10	10
Pigs per pen	15-18	16
Total number of pigs	169	160

C groups: The samples were collected from the control group at the end of the trial; T groups: The samples were collected from the trial group at the end of the trial.

Table S2. Base sequence data information of all samples.

Item	S group	C group	T group
Domain	2.0 ± 0.00	2.0 ± 0.00	2.0 ± 0.00
Phylum	14.0 ± 0.00	14.0 ± 0.00	14.0 ± 0.00
Class	19.0 ± 0.00	19.0 ± 0.00	18.6 ± 0.55
Family	38.4 ± 1.52	40.2 ± 0.34	40.2 ± 0.34
Genus	172.2 ± 2.86[b]	189.8 ± 3.96[a]	191.8 ± 0.84[a]
Species	233.6 ± 3.21[b]	263.2 ± 3.35[a]	265.6 ± 1.34[a]
OTUs	665.0 ± 7.17[b]	706.0 ± 2.55[a]	705.6 ± 6.07[a]

The number of samples was n = 10 (per treatment). Means with different superscripts within a row differ significantly ($p < 0.05$). The significance between mean ± SD was determined separately for each microbial group. S group: The samples were collected from both C and T groups before the trial; C groups: The samples were collected from the control group at the end of the trial; T groups: The samples were collected from the trial group at the end of the trial.

Table S3. Alpha diversity indices of all samples and treatment merged groups.

Item	S group	C group	T group	*p*-value
Observed species	665.0 ± 7.17[b]	706.0 ± 2.55[a]	705.6 ± 6.07[a]	< 0.001
Chao1	693.1 ± 12.06[b]	721.2 ± 7.07[a]	720.7 ± 5.99[a]	< 0.001
Shannon	4.57 ± 0.05[a]	4.17 ± 0.04[b]	4.21 ± 0.06[b]	< 0.001
InvSimpson	31.3 ± 1.37[a]	21.5 ± 0.91[b]	23.4 ± 0.93[b]	< 0.001

The number of samples was n = 10 (per treatment). Means with different superscripts within a row differ significantly ($p < 0.05$). The significance between mean ± SD was determined separately for each microbial group. S group: The samples were collected from both C and T groups before the trial; C groups: The samples were collected from the control group at the end of the trial; T groups: The samples were collected from the trial group at the end of the trial.

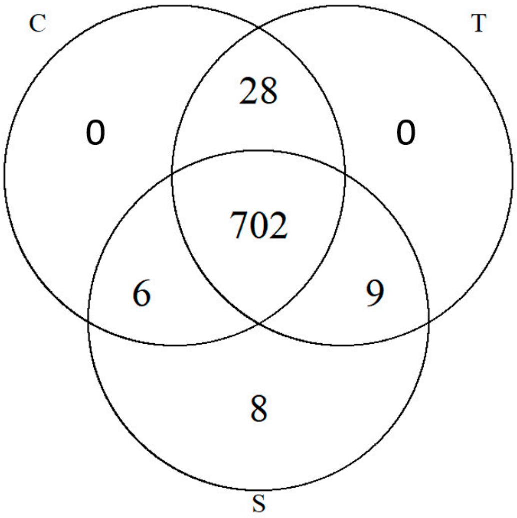

Figure S1. Venn diagram showing the number of unique and shared OTUs of *in silico* merged data of treatments.

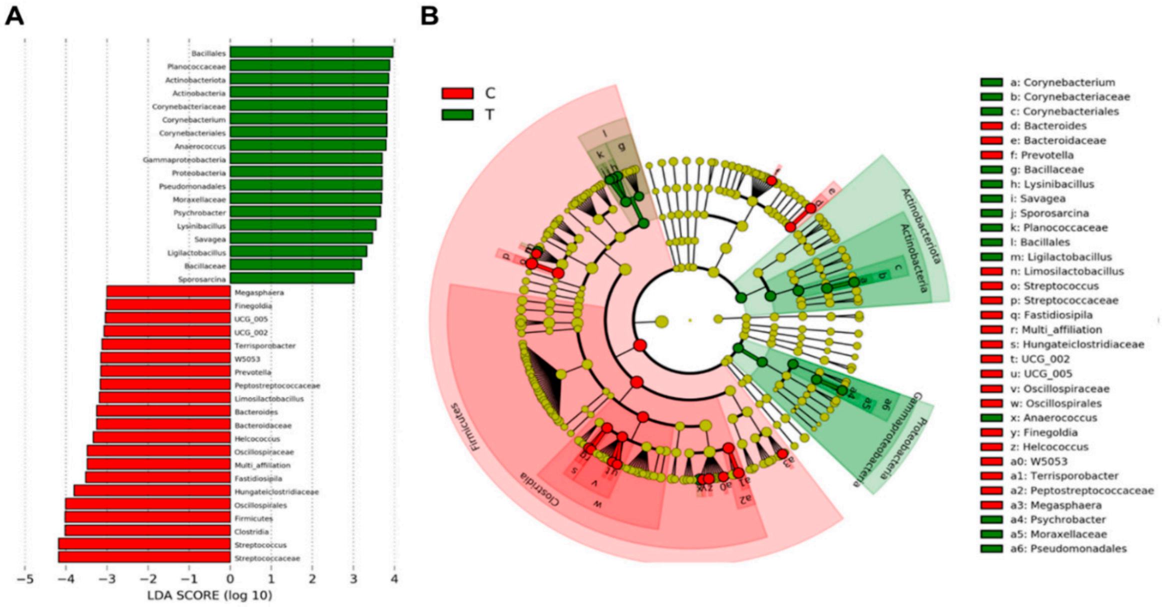

Figure S2. Significantly different abundant taxa between the control (C) and trial (T) groups. (A) Histogram of the results of LEfSe between C and T and their respective effect size (LDA score > 4.0, $p < 0.05$). (B) Cladogram showed a taxonomic representation of differences among treatments.

© 2023 by the authors. Licensee MDPI, Basel, Switzerland. This article is an open access article distributed under the terms and conditions of the Creative Commons Attribution (CC BY) license (https://creativecommons.org/licenses/by/4.0/).
